# Video-assisted thoracoscopic surgery for intrathoracic extramedullary hematopoiesis

**DOI:** 10.4103/0972-9941.30685

**Published:** 2007

**Authors:** E Giblin, K Frankel, K Mortman

**Affiliations:** Department of Surgery, Baystate Medical Center, Springfield, Massachusetts, USA; * Department of Thoracic Surgery, Baystate Medical Center, Springfield, Massachusetts, USA; ** Chief of Thoracic Surgery, Baystate Medical Center, Springfield, Massachusetts, USA

**Keywords:** Hematopoiesis, mediastinal mass, posterior mediastinum, thalassemia, thoracoscopy, thoracotomy

## Abstract

Extramedullary hematopoiesis is a rare cause of an intrathoracic mass in individuals with hemolytic disorders. It can be clinically confused with other tumors of the mediastinum. While radiologic studies often demonstrate findings suggesting intrathoracic extramedullary hematopoiesis, histology is usually required for diagnostic purposes. Thoracotomy was the mainstay procedure for obtaining tissue diagnosis and resection. However, video-assisted thoracoscopy (VATS) is an amendable and less-invasive means of tumor removal. We report a case of a posterior mediastinal extramedullary hematopoietic mass in a forty-two year old male in which VATS was utilized for diagnosis and resection.

## INTRODUCTION

Extramedullary hematopoiesis is a compensatory mechanism in which hematopoietic elements are produced outside of the bone marrow. This occurs in response to an alteration in the normal generation of red bloods cells in the bone marrow, and is most often seen in patients with chronic hemolytic disorders. The exact mechanism for this phenomenon is unknown, but one theory holds that multipotential stem cells undergo hematogenous spread and seed other organs. The liver and spleen, which are part of the reticuloendothelial system, commonly serve as sites for extramedullary hematopoiesis. However, extramedullary hematopoiesis has also been reported in other locations including the lungs, bowel, adrenal glands, dura mater and breast.

Depending on the site of presentation, and the patient's clinical history, sites of extramedullary hematopoiesis may be misinterpreted as a primary malignancy or as metastatic disease. Such misinterpretation may alter a patient's course of therapy and affect prognosis. Extramedullary hematopoiesis presenting as an intrathoracic mass may be deceiving. Although described in the literature, this entity is a rare cause of a mediastinal mass. In patients with a history of chronic benign or neoplastic hemolytic disorders presenting with an intrathoracic mass, a diagnosis of extramedullary hematopoiesis must be considered in the differential diagnosis and diagnostic workup.

## CASE REPORT

**Figure 1:**
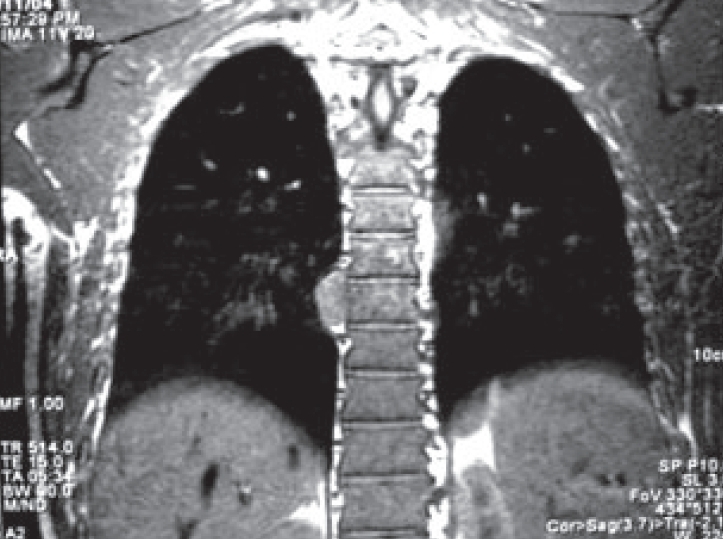
Sagital MRI depicting a right paraspinal mass in the mid thoracic spine

**Figure 2:**
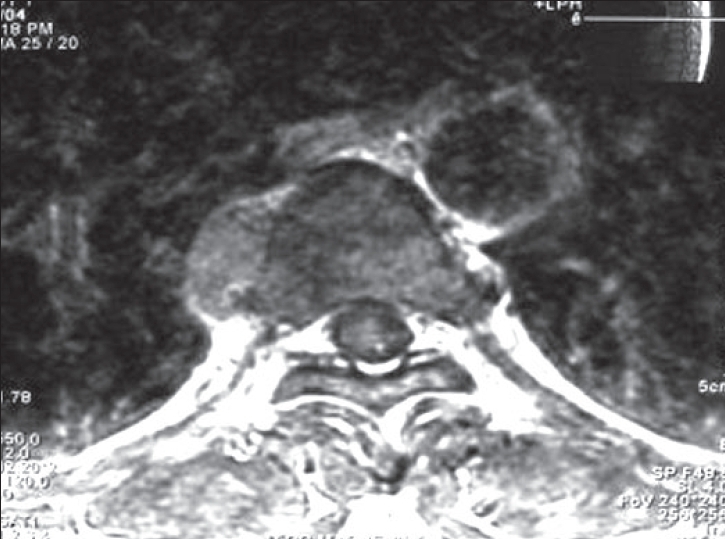
MRI showing a 1.5 × 3.0 cm mass along the lateral margin of the vertebral body. No extension into the neural foramen or spinal canal is demonstrated

A 42-year-old Chinese male with asymptomatic thalassemia minor presented with a non-productive cough of several weeks duration. The patient also had intermittent soreness along the right chest wall in the area of the 7^th^ and 8^th^ ribs radiating from the spine to the anterior axillary line. Medical history was significant for tuberculosis in the past. A chest X-ray demonstrated a posterior mediastinal mass. Follow-up CT scan confirmed a right posterior mediastinal mass along the lateral border of the eighth thoracic vertebral body. MRI showed no evidence of invasion of the mass into the vertebral body or intravertebral foramen [Figures 1 and 2]. Very mild increased uptake of the posterior mediastinal mass was noted on positron emission tomography (PET) scanning.

Neurogenic tumor was suspected and resection of the mediastinal tumor via video-assisted thoracoscopic surgery (VATS) was planned. A 2.5 cm incision in the sixth intercostal space in the midclavicular line and a 5 mm incision in the eighth intercostal space in the midaxillary line were made. Inspection of the right hemithorax revealed extensive adhesions, which were mobilized with sharp dissection and electrocautery. After mobilizing the lung from the paravertebral region, a 2.5 cm cystic mass was encountered. The intercostal vessels supplying the mass were identified, clipped, and divided. The mass was then dissected from the chest wall along with the overlying parietal pleura. Pathological examination of the specimen revealed a 2.2 × 1.2 × 0.7 cm mass consistent with extramedullary hematopoiesis.

## DISCUSSION

Hemolytic conditions can induce the production of blood cells outside of the bone marrow.[1] Common settings in which extramedullary hematopoiesis can be seen include thalassemia, sickle cell disease, spherocytosis, and other hemoglobinopathies.[2] Chronic anemic conditions including pernicious anemia, vitamin B12 deficiency and Gaucher's disease predispose to extramedullary hematopoiesis.[3]

The pathogenesis of this entity remains uncertain. One theory holds that it is due to direct extension from the adjacent intraosseous marrow.[3] Another suggests that extramedullary sites are due to “seeding” from circulatory pluripotent hematopoietic cells.[4] Extramedullary hematopoiesis should be in the differential diagnosis of any chronic hemolytic patient with a mediastinal mass.[5] A close association between this entity and thalassemia has been described.[5] However, case reports have demonstrated this condition in patients without any apparent underlying blood disorder.[1]

Intrathoracic extramedullary hematopoiesis is a rare entity that was first described by Guizetti during an autopsy in 1912.[3] Most cases of thoracic extramedullary hematopoiesis present in adulthood and with a male predominance.[3] Mediastinal extramedullary hematopoiesis in individuals as young as 13 years of age has been reported.[6] These intrathoracic tumors often localize between the 6^th^ and 12^th^ thoracic vertebrae.[1] They can present as unilateral, bilateral or as multiple masses in the paravertebral area, anterior mediastinum, pericardium and pleura.[3,4]

Diagnostic studies including CT scan, MRI, or PET scan can assist in confirming the suspected diagnosis. CT demonstrates well-circumscribed, lobulated soft tissue masses without calcification.[7] MRI assesses the integrity of the adjacent bony cortex and the adipose content of the mass.[3] PET often demonstrates increased uptake in the area of concern.

Treatment depends on the patient's presenting symptoms. If there are no associated adverse symptoms, excision may not be indicated. When symptoms are present, extirpation is indicated. Spinal cord compression can lead to neurogenic symptoms and surgical decompression is essential in this instance. Patients with significant intrathoracic bleeding or with symptoms secondary to space occupying effects of the mass also require surgical extirpation. Extramedullary hematopoietic tissues are known to be radiosensitive and external beam radiation can effectively reduce mass size and associated symptoms.[1]

In cases of extrameduallry hematopoiesis presenting as a mediastinal mass, thoracotomy has been the mainstay procedure in accessing tissue. VATS is a less invasive alternative to thoracotomy for diagnosis and treatment.[3] VATS is especially useful in instances in which the technical approach of a percutaneous biopsy is limited by the location of the mass. It is well known that these tumors are highly vascular and significant bleeding during biopsy or extirpation may be of particular concern.[3,5] VATS allows direct visualization and is a more effective means of controlling hemorrhage compared to the percutaneous approach.[3]
